# Efficacy of different intensities of percutaneous electrolysis for musculoskeletal pain: A systematic review and meta-analysis

**DOI:** 10.3389/fmed.2023.1101447

**Published:** 2023-02-02

**Authors:** Juan Luis Sánchez-González, Víctor Navarro-López, Pablo Cañada-Sánchez, Raúl Juárez-Vela, Regina Ruiz de Viñaspre-Hernández, Sergio Varela-Rodríguez

**Affiliations:** ^1^Department of Nursing and Physiotherapy, University of Salamanca, Salamanca, Spain; ^2^Faculty of Health Sciences, International Doctoral School, Rey Juan Carlos University, Madrid, Spain; ^3^Department of Physical Therapy, Occupational Therapy, Rehabilitation and Physical Medicine, Rey Juan Carlos University, Madrid, Spain; ^4^Faculty of Sport Sciences, Universidad Europea de Madrid, Madrid, Spain; ^5^Faculty of Health Science, University of La Rioja, Logroño, Spain

**Keywords:** percutaneous electrolysis, meta-analysis, musculoskeletal pain, systematic review, physiotherapy

## Abstract

**Objective:**

A meta-analysis of randomized controlled trials (RCTs) was conducted to determine the effect of ultrasound-guided percutaneous electrolysis (PE) alone or as an adjunct to other interventions on pain intensity generated by musculoskeletal disorders, depending on the intensity of the technique.

**Data sources:**

PUBMED, EMBASE, Cochrane Library, Web of Science, SCOPUS, Health Medical Collection, and CINALH from inception to September 2022 were searched to identify documents.

**Study selection:**

Publications investigating the effect of ultrasound-guided PE in musculoskeletal pain.

**Data extraction:**

Data were extracted into predesigned data extraction and tables. Risk of bias was evaluated with the Cochrane Risk of Bias Tool (Rob 2.0). Thirteen articles met inclusion criteria.

**Data analysis:**

Random-effects meta-analysis models were used to quantify the difference in pain between the PE and control groups.

**Data synthesis:**

A significant reduction in pain was found in favor of low- (−1.89; 95% CI: −2.69; −1.10; *p* < 0.001) and high-intensity PE (−0.74; 95% CI: −1.36; −0.11; *p*: 0.02) compared to control group. Low-intensity PE showed significant reduction in pain in the short (−1.73; 95% CI: −3.13; −0.34; *p* < 0.02) and long term (−2.10; 95% CI: −2.93; −1.28; *p* = 0.005), with large effect sizes compared to control group. High-intensity PE only showed significant lower pain than control group in the long term (−0.92; 95% CI: −1.78; −0.07; *p* < 0.03), with a small effect size, but not in the short term.

**Conclusion:**

We found small evidence suggesting that low-intensity PE could be more effective for musculoskeletal pain reduction than high-intensity PE. Nevertheless, scientific evidence on this subject is still scarce and studies comparing the two modalities are warranted.

**Systematic review registration:**

www.crd.york.ac.uk/prospero, identifier CRD42022366935.

## Introduction

Musculoskeletal pain is the clinical entity that generates the most disability, health expenditure and loss of wellbeing in our society. It is estimated that the prevalence may amount to one in two inhabitants in some European populations, being responsible for 49% of absenteeism, in addition to assuming an expenditure between 0.5 and 2.5% of GDP in the countries of the European community (1, 2).

A multimodal approach is recommended to avoid chronification of this condition. Non-pharmacological interventions based on the individual patient context such as therapeutic exercise, pain neuroscience education, and cognitive-behavioral psychological approaches have proven valid in treating persistent musculoskeletal pain (3).

Musculoskeletal pain is classified as primary if it (4) cannot be directly attributed to a known disease or painful process and as secondary if it is caused by a disease that directly affects bones, joints, muscles, and related soft tissues (5). The neural tissue coordinates and unites all these foci of musculoskeletal pain and can also be affected generating neuropathic pain, which in turn can be a focus of musculoskeletal pain (6, 7). Due to the great importance of the nervous system in the management of pain, there are numerous techniques focused on modulating the neuronal electrical component using electric current as a therapeutic physical medium (8).

The most common way to apply electrotherapy to treat musculoskeletal pain is transcutaneous stimulation, which involves the application of a pulsed electrical current across the surface of the skin to potentially activate the underlying nerves, demonstrating short-term effectiveness in reducing musculoskeletal pain (9). In order to be as precise as possible when stimulating the neuromusculoskeletal system, the acupuncture needle began to be used at the beginning of the century as a means to introduce electricity. Due to the great advance in this field in recent decades, many techniques were born and refined, coining in 2003 the concept of invasive physiotherapy by Professor Orlando Mayoral as a subspecialty of physiotherapy (10) to encompass the set of treatment techniques in which the physical agent used is applied percutaneously, that is, through the patient’s skin. Within invasive physiotherapy and thanks to the use of ultrasound to guide its application, much more precise techniques such as percutaneous electrolysis (PE) have been developed.

PE is an invasive physiotherapy technique that consists of the application of a galvanic current through a puncture needle implanted by means of ultrasound support around the lesion, with the proposed objective of generating an analgesic and local inflammatory effect repairing the affected soft tissue (11–13).

More and more clinical trials are trying to investigate the use of PE for the treatment of different musculoskeletal disorders (14–16). The result of these works is the first meta-analysis concerning the effects of PE on pain intensity and disability related to musculoskeletal pain where it is concluded that there is moderate evidence suggesting a large positive effect of PE to reduce pain and moderate evidence of a large decrease in pain-related disability for musculoskeletal pain conditions in the short, medium, and long term (15). However, like other reviews, they add that it is necessary to study the doses to unify more precise criteria for the application of PE and thus be able to reach a consensus on which clinical entities may be the most benefited by this type of therapy (17–20).

Regarding the dosage of PE, Valera-Garrido and Minaya-Muñoz (16) described two modalities according to intensity and time of the application of the galvanic current: high intensity in short times (from 1mA for 3 to 10 s) and low intensity in prolonged times (from 0.3 to 1mA of 50 to 80 s of application).

Therefore, due to the increasing performance of new clinical trials and the need to establish a consensus on how to apply the technique, this systematic review and meta-analysis aims to evaluate the effects of ultrasound-guided PE alone or as an adjunct to other interventions on pain intensity generated by musculoskeletal disorders, depending on the intensity of the technique.

## Methods

### Data source and search methods

Guidelines from the Preferred Reporting Items for Systematic Review and Metaanalysis (PRISMA) statement were consulted to develop this systematic review (21). The computerized databases Medline (Pubmed), SCOPUS, Cochrane Library, Embase, Web of Science, CINAHL, and Health Medical Collection were used to search for relevant studies. Keywords referring to the intervention were used, combined with Boolean operators (complete search strategy is shown in Appendix).

Searches were performed between 12 September-12 October (from the date of inception of each database) using a combination of controlled vocabulary (i.e., medical subject headings) and free-text terms. Search strategies were modified to meet the specific requirements of each database. Hand searches of the reference lists of included studies and previously published systematic reviews were also conducted.

This meta-analysis was registered in the International Prospective Register of Systematic Reviews (PROSPERO registration no.: CRD42022366935).

### Criteria for considering studies and study selection

Studies obtained from the databases were first screened by title and abstract. The screening was performed by two different investigators (SV-R and PC-S) and blinded according to the established inclusion criteria. Discrepancies were resolved by a third investigator (JLS-G).

Inclusion criteria included: randomized clinical trials in English, Spanish, Portuguese, and French that performed an intervention with PE technique and be compared to at least one other group without it. To be considered eligible, studies had to assess pain using standardized scales (VAS, NPRS).

Exclusion criteria included: failure to report variables of interest; non-application of PE; animal studies, systematic reviews, case reports, or meta-analysis.

### Data extraction

A standardized methodology was used to obtain data from studies that met the criteria. Data were obtained on first author, year of publication, design, number of patients, patient demographics, type of device used for intervention, treatment characteristics, and study outcomes (pain). In addition, means and standard deviations of study outcomes were obtained. Authors of included studies were contacted by e-mail, with the aim of accessing possible unclear data. If no response was received, the data were excluded from the analysis.

### Risk of bias and assessment methodological quality of the studies

Two reviewers (VN-L and PC-S) independently assessed risk of bias in the studies and methodological quality of the studies.

The Cochrane Collaboration’s assessment tool was used and consisted of assessment of selection bias, attrition bias, blinding, and sample size (22). This tool evaluates the risk of bias according to 5 domains: randomization process, deviations from intended interventions, missing outcome data, measurement of the outcome, and selection of the reported result. Overall bias was considered as “low risk of bias” if the study was classified as low risk in all domains, “some concerns” if there was at least 1 domain rated as having some concerns, and “high risk of bias” if there was at least 1 domain rated as high risk or several domains rated as having some concerns that could affect the validity of the results.

To analyze the methodological quality of each study, the Physiotherapy Evidence Database (PEDro) was used (23). This scale includes 11 items, with the maximum score being 10, since the first item is not used to calculate the total score, but studies that do not meet this item should be excluded. Scores of 9 and 10 indicate that the studies are of excellent quality, 6–8 of good quality, 4–5 of fair quality, and < 4 of poor methodological quality.

Discrepancies were resolved by a third investigator (JLS-G) throughout the process of analyzing methodological quality and risk of bias.

### Data synthesis and analysis

The quantitative analysis included studies comparing the performance of an intervention with PE technique and control groups using placebo, sham, no intervention, or other active intervention therapies. Differences in pain intensity between the PE and control groups were evaluated. Two main evaluation groups were established, the high-intensity PE group and the low-intensity PE group. When there were several intervention groups, those that applied PE over the tendon were selected.

Pain intensity values, reflected as mean and standard deviation, were used to find the comparison values between the PE group and the control groups. Data regarding pain intensity were collected during muscle contraction using the VAS and NPRS scales. Whenever possible, control groups based on placebo, sham, or no intervention were chosen as comparators. When this was not possible or there were several control groups, those based on active exercise-based interventions were selected. The mean difference between the groups was used to estimate the mean difference, since the measurements were collected in the same unit and with comparable assessments; the means were converted to the standardized mean difference (SMD), with a 95% confidence interval (CI) to obtain the effect size. An effect size of > 0.8 was considered large, between 0.5 and 0.8 was considered medium, and between 0.2 and 0.5 was considered small and *P*-values < 0.05 were considered statistically significant. The degree of heterogeneity between studies was estimated using Cochran’s Q statistical test (with *P*-values < 0.05 considered significant) (24) and the inconsistency index (*I*^2^). An *I*^2^ > 25% was considered to represent small heterogeneity, an *I*^2^ > 50% medium, and an *I*^2^ > 75% large (24). The *I*^2^ is a complement to the *Q*-test, although it has the same power problems when the number of studies is small (24). When the *Q*-test was significant (*P* < 0.1) and/or the *I*^2^ score was > 25%, indicating heterogeneity among studies, the random-effects model was applied in the meta-analysis. A subgroup analysis was performed for each current intensity group, according to measurement time, establishing two subgroups, the immediately post-treatment measurement subgroup, and the post-follow-up measurement subgroup. Asymmetry was assessed using a funnel plot in those analyses consisting of at least five studies, indicating the possible risk of publication of small studies with negative results. The studies were analyzed with Review Manager 5.3 statistical software.

### Interrater reliability

Interrater reliability for screening, data extraction, risk of bias assessment, and quality of the evidence rating was assessed using percentage agreement and Cohen’s kappa coefficient (23, 24). There was strong agreement between reviewers for the screening records and full texts (91% agreement rate and *k* = 0.91), the data extraction process (91% agreement rate and *k* = 0.91), the risk of bias assessment (92% agreement rate and *k* = 0.82) and the quality and strength of the evidence assessment (94% rate and *k* = 0.85) (24, 25).

### Quality of evidence

The Grading of Recommendations Assessment, Development and Evaluation (GRADE) (26) approach was used to evaluate the quality of evidence for the PE technique. It was carried out independently by two authors and in case of discrepancies a third author acted.

The quality of evidence was classified as high, moderate, low, or very low according to the presence of study limitations (RoB), inconsistency of results, unexplained heterogeneity, imprecision of results, high probability of publication bias, or lack of directionality of evidence (27). The quality of evidence was classified as very low when all items had a serious risk or more than two items had a very serious risk; low when two or three items had a serious risk or one or two items had a very serious risk; low when two or three items had a serious risk or one or two items had a very serious risk; moderate when one item included a serious risk; high when all items were negative.

## Results

**FIGURE 1:**
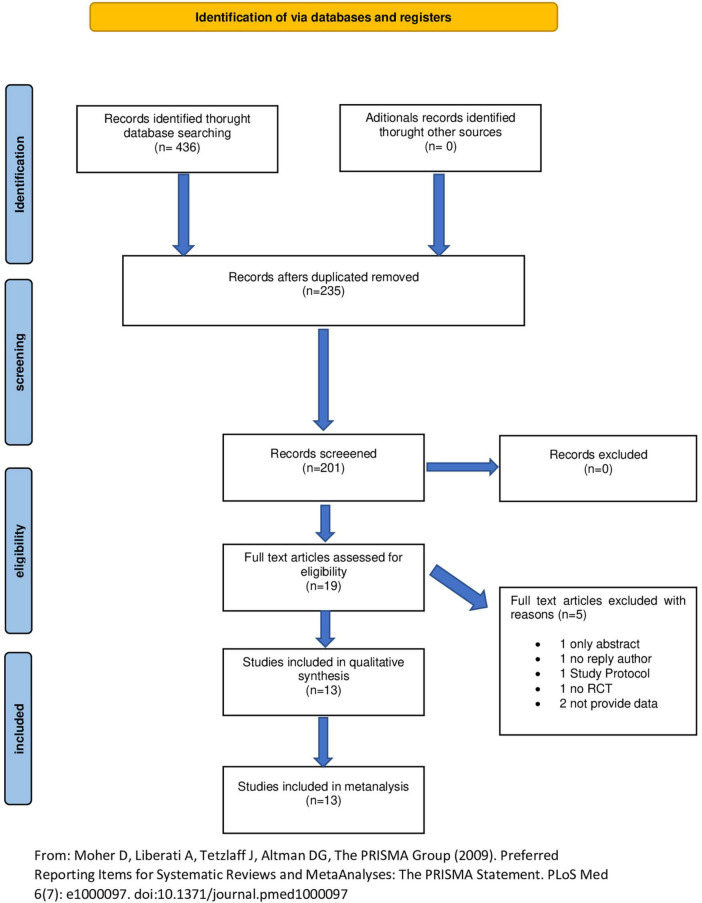
Identification of *via* databases and registers. Adapted from Moher et al. (21).

The search found 436 records, of which 235 were duplicates and 201 were screened by title and abstract. 19 studies were potentially relevant and full reports obtained and screened. 6 studies were excluded with reasons. Finally, 13 RTCs met the eligibility criteria and were included for qualitative and quantitative analysis with a total of 673 subjects (15, 28–38). The whole screening process is shown in the PRISMA flow diagram (Figure 1).

### Characteristics of included studies

The characteristics of the participants in the included studies are detailed in Table 1. All included studies applied PE; 10 also applied another type of treatment together: 1 applied PE and ultrasound (28) 7 applied PE combined with exercise (15, 30, 31, 34–37), 1 applied PE combined with exercise and manual therapy (32). The type of intensity applied was high in 7 studies (14, 28, 33, 34, 36–38), and low in 6 studies (15, 29–32, 35). The type of comparison was heterogeneous, 1 compared with dry needling (28), 1 with ultrasound (29), 1 with manual therapy and exercise (32), 1 with sham, 1 with no intervention (33), 1 with active program (34), 1 with conventional physiotherapy (14), 2 with dry needling combined with exercise (31, 35), 3 with exercise (30, 38), 3 with sham PE and exercise (15, 36, 37).

**TABLE 1:** Participant characteristics.

Study	Design	Group (sample size)	Gender, male (female)	Age, years	Pain localization	Pain duration (months)	Stimulation protocol	Pain outcome
Al-Boloushi et al. (28)	RCT	G1 (51)	15 (36)	50 ± 9	Plantar heel pain	6.0 ± 6.0	TrP dry needling involved in plantar heel pain (5 s 1 Hz/s): soleus, gastrocnemius, quadratus plantae, flexor digitorum brevis, and abductor hallucis. 1/week over 4 weeks.	VAS
		G2 (51)	15 (36)	48 ± 9		9.9 ± 11.5	Trp PE 1.5 mA (same procedure with galvanic current) 1/week over 4 weeks.	
de la Barra-Ortiz et al. (29)	RCT	G1 (24)	11 (13)	23 ± 2	Myofascial pain	NR	Ultrasound 15 min + PE on TrP trapezius (3 impacts 0.6 mA)	VAS
		G2 (24)	12 (12)	22 ± 2		NR	Ultrasound 15 min 1 session	
Arías-Buría et al. (30)	RCT	G1 (17)	4 (13)	58 ± 7	Shoulder pain	11.2 ± 2.7	PE 1/week over 4 weeks (0.350 mA 1, 2 min in supraspinatus tendon) + Eccentric exercises	NPRS-11
		G2 (19)	5 (14)	57 ± 6		10.6 ± 2.6	Eccentric exercise 2/day over 4 weeks (3 exercises 3 × 10)	
Fernández-Rodríguez et al. (15)	RCT	G1 (38)	15 (23)	45 ± 11	Plantar Heel pain	>3	PE 1/week over 5 weeks (28 mC in proximal plantar fascia; intensity is not specified) + exercise	NPRS-11
		G2 (29)	10 (19)	47 ± 11		>3	sham PE 1/week over 5 weeks (without current) + exercise (not specified)	
Rodríguez-Huguet et al. (31)	RCT	G1 (18)	11 (7)	41 ± 8	Shoulder pain	NR	Dry needling on TrP of supraspinatus muscle 1/week over 4 weeks + exercise 1/day	NPRS-11
		G2 (18)	16 (2)	39 ± 11		NR	PE on supraspinatus tendon (0.350 mA 1.2 min) 1/week over 4 weeks + exercise 1/day (3 exercises 3 × 10)	
Rodríguez-Huguet et al. (35)	RCT	G1 (16)	10 (6)	40 ± 16	Lateral elbow pain	NR	PE on epicondyle tendon (0.350 mA, 1.2 mA) 1/week over 4 weeks + eccentric exercise 2/day (3 × 10)	NPRS-11
		G2 (16)	10 (6)	36 ± 12		NR	Dry needling on epicondylar musculature 1/week over 4 weeks + eccentric exercise 2/day (3 × 10)	
López-Royo et al. (36)	RCT	G1 (16)	13 (3)	33 ± 8	Patellar tendinopathy	19 ± 28.4	Dry needling on patellar tendon 1 every 2 weeks over 8 weeks + exercise 2/day	VAS
		G2 (16)	14 (2)	31 ± 7		13.9 ± 10.3	PE on patellar tendon (3 impacts of 3 mA 3 s) 1 every 2 weeks over 8 weeks + exercise 2/day	
		G3 (16)	15 (1)	33 ± 6		18.4 ± 16.6	Sham needling procedure 1 every 2 weeks over 8 weeks (without introducing the needle?) + exercise (3 × 15 single leg squat) 2/day	
López-Martos et al. (37)	RCT	G1 (20)	5 (15)	38.5 ± (18–57) IQR	Temporomandi bular pain	>6	PE on lateral pterygoid muscle 1/week over 3 weeks (3 impacts of 2 mA 3 s) + exercise 2 week after intervention (masticatory muscles)	VAS
		G2 (20)	2 (18)	36 ± (19–58) IQR		>6	Deep dry needling on lateral pterygoid muscle 1/week over 3 weeks (without current) + exercise	
		G3 (20)	1 (19)	42 ± (25–62) IQR		>6	Sham PE 1/week over 3 weeks (pressure with plastic protective tube without introducing the needle) + exercise	
de Miguel-Valtierra et al. (32)	RCT	G1 (25)	12 (13)	55 ± 11	Shoulder pain	11.2 ± 10.6	Manual therapy (joint mobilizations and soft tissues techniques) + Exercise (3 × 12 of 3 exercises). 1/week over 5 weeks	NPRS-11
		G2 (25)	11 (14)	55 ± 14		12.6 ± 14.4	PE on supraspinatus tendon (0.350 mA 1.2 min) + Manual therapy + Exercise. 1/week over 5 weeks	
Dolores R-Moreno (33)	RCT	G1 (10)	NR	40 ± 4	Shoulder pain	>3	Control group without intervention	VAS
		G2 (10)	NR	40 ± 3		>3	PE in trigger points 1/week over 3 weeks (3 impacts of 6 mA during 4 s)	
		G3 (10)	NR	40 ± 4		>3	PE in infraspinatus tendon 1/week over 3 weeks (3 impacts of 6 mA during 4 s)	
		G4 (10)	NR	40 ± 5		>3	PE in both locations 1/week over 3 weeks (3 impacts of 6 mA during 4 s)	
Moreno et al. (34)	RCT	G1 (11)	11 (0)	27 ± 5	Groin pain	1: 5 1–2.5: 4 2.5–6: 2	PE 2 per week during phase 1 of active physical therapy program (3 impacts of 3 mA 5 s) on adductor longus tendon + active physical therapy program	NPRS-11
		G2 (13)	13 (0)	25 ± 5		1: 6 1–2.5: 3 2.5–6: 3 >6: 1	Active physiotherapy program (3 phases depending on symptomatology; at least 1 week in each phase)	
García-Naranjo et al. (14)	RCT	G1 (50)	20 (30)	35 ± 8	Whiplash associated pain	<1	Standard physiotherapy 5/week over 4 weeks (microwave 10 min, TENS 5–10 min, pulsed US 10 min, exercises 20 min)	VAS
		G2 (50)	16 (34)	41 ± 9		<1	PE on levator scapulae 1/week over 3 weeks (3 punctures with 1–2 min rest between them; starting at 2 mA, increasing 1 mA/s to reach 4 mA and stopping at that moment)	
De-la-Cruz-Torres et al. (38)	RCT	G1	1 (9)	20 ± 3	Chronic soleus injury	>6	PE on soleus muscle (3 impacts of 2.5 mA 3 s) 1/week for 2 weeks	NPRS-11
		G2	1 (9)	21 ± 3		>6	Eccentric exercise of soleus muscle (3 × 15) one daily, 4 days/week for 4 weeks	
		G3	1 (9)	21 ± 3		>6	PE + eccentric exercise	

NPRS, numerical pain rating scale; VAS, visual analog scale; RCT, randomized controlled trial.

The musculoskeletal conditions were heterogeneous including plantar heel pain (15, 28) pain in myofascial trigger points (29), shoulder pain (30, 33), groin pain (34), lateral epicondylalgia (35), patellar tendinopathy (36), temporo-mandibular pain (37), whiplash-associated pain (14), and chronic soleus injury (38). All trials applied PE, but there was higher diversity in terms of protocol. The number of sessions were 5 in 2 studies (15, 32), 4 in 5 studies (28, 30, 31, 35, 36), 3 in 3 studies (14, 33, 37), 2 in 2 studies (34, 38), and 1 in 1 studies (29). The frequency of sessions were 1/week in 10 studies (14, 15, 28–31, 33, 35, 37, 38), 2/week in 1 study (34), and 1/2 weeks in 1 study (36). The intensity of the electrical current, the time of electrical current, gauge, depth, or device were detailed in Table 1. The PE parameters applied in each trial were summarized in Supplementary Table 1.

### Quality assessment

Methodological quality scores ranged from 4 to 10 out of a maximum of 10 points. 10 studies (76%) were of high methodological quality (greater than or equal to 6 points). Table 2 lists the details of the PEDro scale.

**TABLE 2:** Methodological score of randomized clinical trials using the Physiotherapy Evidence Database (PEDro) scale.

Study	1	2	3	4	5	6	7	8	9	10	Total
Al-Boloushi et al. (28)	Y	Y	Y	N	N	Y	Y	Y	Y	Y	8
de la Barra-Ortiz et al. (29)	Y	Y	Y	Y	Y	Y	Y	Y	Y	Y	10
Arías-Buría et al. (30)	Y	Y	Y	Y	N	N	N	Y	Y	Y	7
Fernández-Rodríguez et al. (15)	Y	N	Y	Y	Y	N	Y	N	Y	Y	7
Rodríguez-Huguet et al. (31)	Y	Y	Y	N	N	Y	Y	N	Y	Y	7
Rodríguez-Huguet et al. (35)	Y	Y	Y	N	N	Y	Y	N	Y	Y	7
López-Royo et al. (36)	Y	N	N	N	N	Y	Y	Y	N	Y	5
López-Martos et al. (37)	Y	N	Y	Y	N	N	Y	Y	Y	Y	7
de Miguel-Valtierra et al. (32)	Y	Y	Y	Y	N	Y	Y	Y	Y	Y	9
Dolores R-Moreno (33)	N	N	Y	N	N	N	Y	Y	Y	Y	5
Moreno et al. (34)	Y	Y	Y	N	N	Y	Y	N	Y	Y	7
García-Naranjo et al. (14)	Y	Y	Y	N	N	Y	Y	Y	Y	Y	8
De-la-Cruz-Torres et al. (38)	N	N	Y	N	N	N	N	Y	Y	Y	4

Y > yes; N > no. (1): random allocation of participants; (2): concealed allocation; (3): similarity between groups at baseline; (4): participant blinding; (5): therapist blinding; (6): assessor blinding; (7): dropout rate less than 15%; (8): intention-to-treat analysis; (9): between-group statistical comparisons; (10): point measures and variability data.

### Risk of bias

**FIGURE 2:**
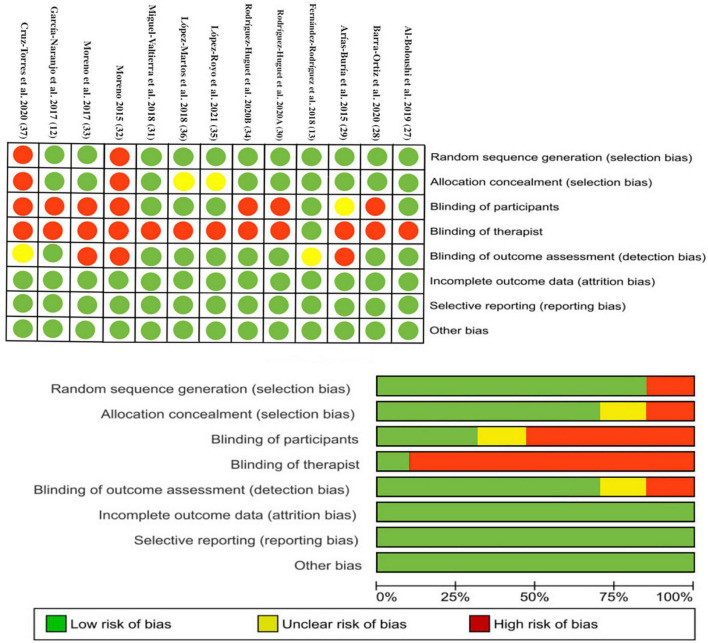
Plots of risk of bias of the included studies.

As assessed by the Cochrane Collaboration’s RCT tool, 92% of the studies showed high risk of bias in blinding of the therapist, 53% showed high risk in blinding of participants, 23.07% showed high risk in blinding of outcome assessment, and 15.38% showed high risk in random sequence generation, and allocation concealment. The details of the risk-of-bias assessment of the included trials are shown in Figure 2.

### Effects of intervention

#### Effects of low intensity percutaneous electrolysis on pain

**FIGURE 3:**
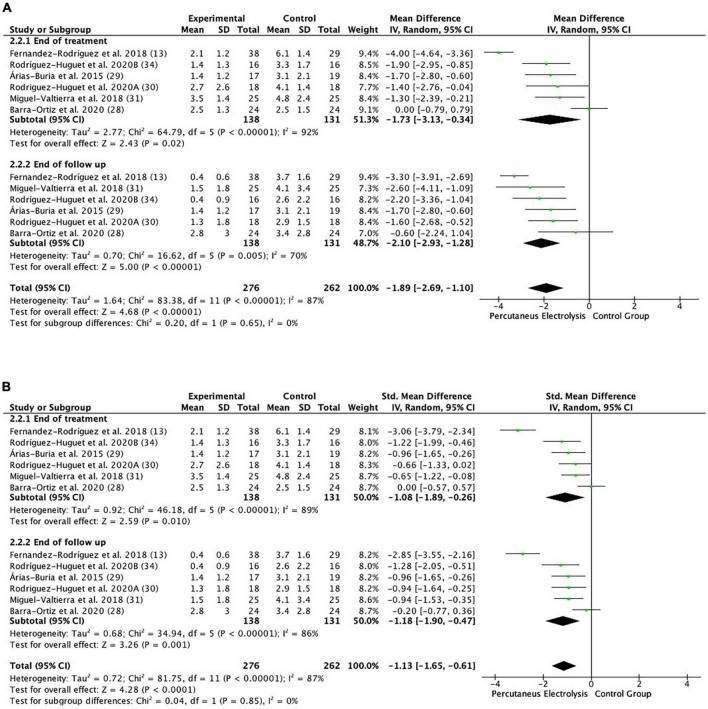
Forest plot of the results of a random-effects meta-analysis shown as **(A)** mean differences (MD) and **(B)** SMD, with 95% confidence interval (CI) for the comparison of mean pain in the low intensity PE group and the control group. Short-term and long-term subgroups are reflected, depending on the time of the pain assessment. The shaded square represents the point estimate for each individual study and the weight of the study in the meta-analysis. The diamond represents the overall mean difference of the studies.

**FIGURE 4:**
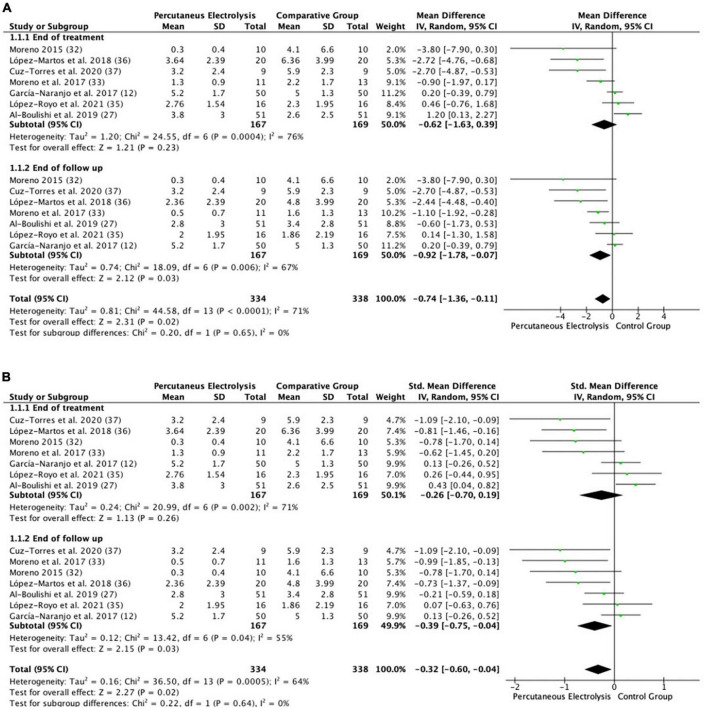
Forest plot of the results of a random-effects meta-analysis shown as **(A)** mean differences (MD) and **(B)** SMD, with 95% confidence interval (CI) for the comparison of mean pain in the high intensity PE group and the control group. Short-term and long-term subgroups are reflected, depending on the time of the pain assessment. The shaded square represents the point estimate for each individual study and the weight of the study in the meta-analysis. The diamond represents the overall mean difference of the studies.

The meta-analysis showed that significantly (*p* < 0.001), the PE group showed a lower mean pain of 1.89 points, than the control intervention group (MD: −1.89; 95% CI: −2.69; −1.10; *Z*: 4.68; *p* < 0.001; *I*^2^: 87%), with a large effect size (SMD: −1.13; 95% CI: −1.65; −0.61; *Z*: 4.28; *p* < 0.001; *I*^2^: 87%) (Figures 3, 4). Between-study heterogeneity was high (*I*^2^: 87%). Subgroup analysis showed no differences (*p* = 0.65) between pain assessment times, with significantly lower pain in the PE intervention group immediately after treatment (MD: −1.73; 95% CI: −3.13; −0.34; *Z*: 2.43; *p* < 0.02; *I*^2^: 92%), with a large effect size (SMD: −1.08; 95% CI: −1.89; −0.26; *Z*: 2.59; *p*: 0.01; *I*^2^: 89%), and at the end of the follow-up time (MD: −2.10; 95% CI: −2.93; −1.28; *Z*: 5; *p* = 0.005; *I*^2^: 70%), with a large effect size (SMD: −1.18; 95% CI: −1.90; −0.47; *Z*: 3.26; *p*: 0.001; *I*^2^: 86%). The funnel plot presents asymmetry, indicating the risk of publication bias (Supplementary Figure 1). Low intensity PE funnel plot. Dispersion of effect sizes. *X*-axis: observed effect sizes. *Y*-axis: inversed standard error (higher values on the *Y*-axis represent lower standard errors). Slight asymmetry, meaning possible publication bias.

#### Effects of high intensity percutaneous electrolysis on pain

The meta-analysis showed that significantly (*p* < 0.02), the PE intervention group showed a lower mean pain of 0.74 points, than the control intervention group (MD: −0.74; 95% CI: −1.36; −0.11; *Z*: 2.31; *p*: 0.02; *I*^2^: 71%), with a small effect size (SMD: −0.32; 95% CI: −0.60; −0.04; *Z*: 2.27; *p*: 0.02; *I*^2^: 64%). Between-study heterogeneity was high (*I*^2^: 64%). Subgroup analysis showed that there were non-significant differences (*p* = 0.64) between pain assessment times. Immediately after the end of the intervention, no significant differences were observed between groups (MD: −0.62; 95% CI: −1.63; 0.39; *Z*: 1.21; *p*: 0.23; *I*^2^: 76%), but significant differences between groups were observed at the end of the follow-up time, showing a lower mean pain of 0.92 points, in the PE group than in the control intervention group (MD: −0.92; 95% CI: −1.78; −0.07; *Z*: 2.12; *p* < 0.03; *I*^2^: 67%), with a small effect size (MD: −0.39; 95% CI: −0.75; −0.04; *Z*: 2.15; *p* < 0.04; *I*^2^: 64%). The funnel plot presents asymmetry, indicating the risk of publication bias (Supplementary Figure 2). High intensity PE funnel plot. Dispersion of effect sizes. *X*-axis: observed effect sizes. *Y*-axis: inversed standard error (higher values on the *Y*-axis represent lower standard errors). Slight asymmetry, meaning possible publication bias.

### Quality of evidence (GRADE)

Table 3 collects the details of the GRADE assessment, showing the risk of bias, inconsistency of results, indirect evidence, imprecision of results, and high probability of publication bias. Serious inconsistency of results (heterogeneity) and risk of bias were downgraded to a small level of evidence for the overall effect of ultrasound-guided PE at both high and low intensities for pain.

**TABLE 3:** GRADE evidence for percutaneous electrolysis to treat pain for musculoskeletal pain conditions.

Number of studies	Risk of bias*	Inconsistency^†^	Indirectness^‡^	Imprecision^§^	Publication bias^¶^	MD or SMD (95% CI)	Quality of evidence
**Low-intensity percutaneous electrolysis**							
Six trials (*n* = 538)	Serious (mainly by blinding the therapist)	Very serious (*I*^2^ = 87%)	No serious	No serious	No serious	MD = −1.89 (−2.69, −1.10) SMD = −1.13 (−1.65, −0.61)	Small
**High-intensity percutaneous electrolysis**							
Seven trials (*n* = 672)	Serious (mainly by blinding the therapist)	Serious (*I*^2^ = 71%)	No serious	No serious	No serious	MD = −0.74 (−1.36, −0.11) SMD = −0.32 − 0.60, −0.04)	Small

GRADE, Grading of Recommendations Assessment, Development and Evaluation; MD, mean difference; SMD, standardized mean difference.

*“No,” most information is from results at low risk of bias; “serious,” crucial limitation for one criterion or some limitations for multiple criteria sufficient to lower confidence in the estimate of effect; “very serious,” crucial limitation for one or more criteria sufficient to substantially lower confidence in the estimate of effect.

^†^“Serious,” *I*^2^ > 40%; “very serious,” *I*^2^ > 80%.

^‡^No indirectness of evidence was found in any study.

^§^ Based on sample size. “Serious,” *n* < 250 subjects; “very serious,” *n* < 250 and the estimated effect is little or absent.

^¶^ Based on funnel plots. No publication bias was found. Funnel plots are not shown because the number of trials was less than 10.

## Discussion

This meta-analysis aimed to investigate the effects of different intensities in the application of ultrasound-guided PE on the management of musculoskeletal pain. The results found that low-intensity PE showed a large effect size on pain relief in comparison to control groups, both after the treatment and at the end of follow-up. Meanwhile, high-intensity PE had a small effect size on pain reduction at the end of follow-up but no significant differences were observed immediately after the treatment. The level of evidence (GRADE) was small due to high heterogeneity and high risk of bias related to blinding.

Four trials involved at least one group receiving PE alone (14, 28, 34, 38), while the rest of the studies combined PE with another intervention such as exercise, ultrasound or manual therapy (15, 29–33, 35–37). Therefore, most of the included studies could have considered PE as part of a multimodal treatment, which reproduces with greater plausibility a real clinical scenario, but at the same time, the percentage of the change in pain that can be attributed to the technique cannot be reliably estimated. In our opinion, both combining PE with another intervention and applying it alone are appropriate methods in research to continue growing the scientific evidence on PE. On the other hand, all studies compared PE with another intervention (14, 15, 28–32, 34–38) the exception of the one conducted by Moreno (33), which compared it with a non-intervention control group. In addition, all trials used the VAS and NPRS scales to assess pain outcomes, whereas questionnaires assessing functionality and other variables were not included in the present meta-analysis due to the large variability.

Looking at a general overview of our results, we found that EP is effective in the management of musculoskeletal disorders, which is consistent with the conclusions of the meta-analysis conducted by Gómez-Chiguano et al. (17) and other qualitative systematic reviews (18, 19, 39, 40). These articles reflected that one limitation of the scientific evidence concerning PE is the high variability in the parameters of application of the galvanic current. For this reason, the present meta-analysis has investigated possible differences in pain outcomes based on electric current intensity. The results indicate that both low-intensity and high-intensity PE modalities seem to be effective for treating musculoskeletal disorders, but greater pain reduction was obtained with the application of low-intensity PE than high-intensity PE (both compared to control groups). This could lead clinicians to select a low-intensity treatment modality (<1 mA), which is usually associated with longer application times (50–80 s) (16). Most of the low-intensity PE studies included in this meta-analysis performed 0.350 mA for 72 s. In addition, it should be noted that this modality is commonly better tolerated by the patient.

Salaffi et al. (41) estimated the minimal clinically important difference (MCID) as a reduction of 1 point or 15% from baseline scores for NPRS in patients with musculoskeletal pain. Low-intensity PE also benefited from the MCID study, as it exceeded this value by almost one point of pain decrease (−1.84), whereas high-intensity PE did not reach this threshold (−0.74).

As mentioned above, the choice of the dosage of administration of PE therapy is poorly supported by scientific evidence. To the best of our knowledge, only a pilot study has compared two PE protocols in patients, finding no differences between them in sensitivity and pain associated with patellofemoral pain syndrome (42). In this case, the two protocols presented little variation in the intensity parameter (0.220 mA for 30 s and 0.660 mA for 10 s) and both would belong to the low-intensity modality. Additionally, Varela-Rodríguez et al. (43) conducted a randomized controlled trial in healthy subjects and also observed no differences between low- and high-intensity PE (0.3 mA for 90 s and 3 mA for 9 s) in most of the included variables related to endogenous pain modulation. However, the results of this meta-analysis seem to favor low-intensity modality, contrasting with the limited differences found in the two articles cited previously. Due to the scarcity of publications exploring PE dosage in depth, further research comparing different PE protocols is required.

Regarding other techniques employing electric current through needles, such as electroacupuncture, better results were observed with the use of high-intensity current in alleviating pain intensity and increasing conditioned pain modulation in patients with knee osteoarthritis (44). These results are conflicting with ours and the authors provide the possible explanation that high-intensity electroacupuncture stimulates Aδ and/or C fibers and may activate conditioned pain modulation, while low-intensity electroacupuncture mainly stimulates Aβ fibers and only enable the gate control mechanism. However, low- and high-intensity PE showed no difference in conditioned pain modulation in healthy subjects, at least in the short term (43). This could indicate that PE and electroacupuncture have different mechanisms of pain relief and could be a justification for the discrepancy in results.

Even though most of the included studies demonstrated a high methodological quality, the results should be interpreted cautiously due to the high risk of bias on “blinding the therapist” domain. Only the study conducted by Fernández-Rodríguez et al. (15) blinded the therapist who performed the needling intervention, through the involvement of a second investigator who selected the parameters of the galvanic current and turned off the ultrasonographic monitor, so the clinical researcher was unable to see the hydrogen gas produced by the electrolytic reaction. In addition, the difficulty of blinding participants was present in almost half of the selected studies, mainly related to the nature of the intervention and the lack of inclusion of a sham group as comparison (14, 29, 31, 33–35, 38).

This meta-analysis had several strengths, such as blind peer screening of studies, systematic and transparent review of literature, assessment of risk of bias and methodological quality, and inclusion of randomized controlled trials (RCTs). Furthermore, this is the first meta-analysis exploring the effects of different intensities of PE on pain in patients with musculoskeletal disorders.

However, some limitations must be acknowledged. Firstly, the quality of most of the included studies was compromised by high heterogeneity and risk of bias in certain domains. Additionally, the dosage of PE presented a substantial variability, especially in the high-intensity modality, showing a lack of consensus on the application parameters. Finally, apart from the small number of studies included (*n* = 13), a large heterogeneity in the pathologies studied was observed, involving very different indications within the field of musculoskeletal disorders (e.g., tendinopathies, whiplash syndrome, temporo-mandibular pain, or chronic muscle injury).

## Conclusion

This meta-analysis found small evidence suggesting a large effect of low-intensity PE for decreasing musculoskeletal pain in comparison to control groups, both after the treatment and at the end of follow-up. Meanwhile, high-intensity PE showed a small effect size on pain reduction at the end of follow-up, with no differences immediately after treatment. This could indicate a greater effectiveness in the management of musculoskeletal pain of the low-intensity modality, but further research is needed to determine the appropriate parameters of application of the technique.

## Data availability statement

The original contributions presented in this study are included in the article/Supplementary material, further inquiries can be directed to the corresponding author.

## Author contributions

JLS-G designed the study, participated in the research, and drafted the manuscript. VN-L participated in the operation, drafted the manuscript, collected the data, and performed the analysis. PC-S participated in the operation and drafted the manuscript. RJ-V and RRV-H participated in the operation and revised the article. SV-R designed the study, participated in the research, and drafted the manuscript. All authors contributed to the article and approved the submitted version.

## Supplementary material

Supplementary Figure 1 Funnel low.

Supplementary Figure 2 Funnel high.

Supplementary Table 1 Parameters of percutaneous electrolysis interventions of the included trials.

## Appendix

**Appendix 1:** Database formulas during literature search.

**PubMed Search Formula: 55**

(“Percutaneous electrolysis” OR “Percutaneous needle electrolysis” OR “needle percutaneous electrolysis” OR “galvanic electrolysis” OR “intratissue percutaneous electrolysis” OR (“percutaneous” AND “electrolysis”).

**CINAHL/Medline Search Formula (EBSCO)**

(“ultrasound-guided percutaneous electrolysis” OR “percutaneous electrolysis” OR “needle percutaneous electrolysis” OR “percutaneous needle electrolysis” OR (“percutaneous” AND “electrolysis”) OR “intratissue percutaneous electrolysis” OR “ultrasound guided galvanic electrolysis”.

**PEDro Search Formula**

Abstract & Title: Percutaneous Electrolysis Method: Clinical trial When Searching: AND.

**Cochrane Library Search Formula**

Percutaneous electrolysis OR Percutaneous needle electrolysis OR needle percutaneous electrolysis OR galvanic electrolysis OR intratissue percutaneous electrolysis in Title Abstract Keyword AND percutaneous in Title Abstract Keyword AND electrolysis in Title Abstract Keyword.

**SCOPUS Search Formula**

TITLE-ABS-KEY (“Percutaneous electrolysis” OR “Percutaneous needle electrolysis” OR “needle percutaneous electrolysis” OR “galvanic electrolysis” OR “intratissue percutaneous electrolysis”) OR (“percutaneous” AND “electrolysis”).

**WOS Search Formula**

(“Percutaneous electrolysis” OR “Percutaneous needle electrolysis” OR “needle percutaneous electrolysis” OR “galvanic electrolysis” OR “intratissue percutaneous electrolysis” OR (“percutaneous” AND “electrolysis”).

**Embase Search Formula**

**Health & Medical Collection**

(“Percutaneous electrolysis” OR “Percutaneous needle electrolysis” OR “needle percutaneous electrolysis” OR “galvanic electrolysis” OR “intratissue percutaneous electrolysis” OR (“percutaneous” AND “electrolysis”).

## References

[1] Bonanni R Cariati I Tancredi V Iundusi R Gasbarra E Tarantino U. Chronic pain in musculoskeletal diseases: do you know your enemy? *J Clin Med.* (2022) 11:2609. 10.3390/jcm11092609 35566735 PMC9101840

[2] Bevan S. Economic impact of musculoskeletal disorders (MSDs) on work in Europe. *Best Pract Res Clin Rheumatol.* (2015) 29:356–73. 10.1016/j.berh.2015.08.002 26612235

[3] Cohen S Vase L Hooten W. Chronic pain: an update on burden, best practices, and new advances. *Lancet.* (2021) 397:2082–97. 10.1016/S0140-6736(21)00393-7 34062143

[4] Cimmino M Ferrone C Cutolo M. Epidemiology of chronic musculoskeletal pain. *Best Pract Res Clin Rheumatol.* (2011) 25:173–83. 10.1016/j.berh.2010.01.012 22094194

[5] Perrot S Cohen M Barke A Korwisi B Rief W Treede R. The IASP classification of chronic pain for ICD-11: chronic secondary musculoskeletal pain. *Pain.* (2019) 160:77–82. 10.1097/j.pain.0000000000001389 30586074

[6] Bouhassira D. Neuropathic pain: definition, assessment and epidemiology. *Rev Neurol (Paris).* (2019) 175:16–25. 10.1016/j.neurol.2018.09.016 30385075

[7] Colloca L Ludman T Bouhassira D Baron R Dickenson A Yarnitsky D Neuropathic pain. *Nat Rev Dis Prim.* (2017) 3:17002. 10.1038/nrdp.2017.2 28205574 PMC5371025

[8] Knotkova H Hamani C Sivanesan E le Beuffe M Moon J Cohen S Neuromodulation for chronic pain. *Lancet.* (2021) 397:2111–24. 10.1016/S0140-6736(21)00794-7 34062145

[9] Johnson M Paley C Jones G Mulvey M Wittkopf P. Efficacy and safety of transcutaneous electrical nerve stimulation (TENS) for acute and chronic pain in adults: a systematic review and meta-analysis of 381 studies (the meta-TENS study). *BMJ Open.* (2022) 12:e051073. 10.1136/bmjopen-2021-051073 35144946 PMC8845179

[10] del Moral O. Fisioterapia invasiva del síndrome de dolor miofascial. *Fisioterapia.* (2005) 27:69–75. 10.1016/S0211-5638(05)73419-2

[11] Martin Urrialde H. Invasive methods: percutaneous electrolysis intratissue and mesotherapy. 1st ed. In: Seco Calvo J editor. *Methods of Intervention in Physiotherapy.* (Madrid: Editorial Médico Panamericana) (2015). p. 215–23.

[12] Abat F Gelber P Polidori F Monllau J Sanchez-Ibañez J. Clinical results after ultrasound-guided intratissue percutaneous electrolysis (EPI^®^) and eccentric exercise in the treatment of patellar tendinopathy. *Knee Surg Sports Traumatol Arthrosc.* (2015) 23:1046–52. 10.1007/s00167-014-2855-2 24477495

[13] Valera-Garrido F Minaya-Muñoz F Medina-Mirapeix F. Ultrasound-guided percutaneous needle electrolysis in chronic lateral epicondylitis: short-term and long-term results. *Acupunct Med.* (2014) 32:446–54. 10.1136/acupmed-2014-010619 25122629 PMC4283658

[14] García Naranjo J Barroso Rosa S Loro Ferrer J Limiñana Cañal J Suarez Hernández E. A novel approach in the treatment of acute whiplash syndrome: ultrasound-guided needle percutaneous electrolysis. A randomized controlled trial. *Orthop Traumatol Surg Res.* (2017) 103:1229–34. 10.1016/j.otsr.2017.09.012 28987529

[15] Fernández-Rodríguez T Fernández-Rolle Á Truyols-Domínguez S Benítez-Martínez J Casaña-Granell J. Prospective randomized trial of electrolysis for chronic plantar heel pain. *Foot Ankle Int.* (2018) 39:1039–46. 10.1177/1071100718773998 29771148

[16] Valera-Garrido F Minaya-Muñoz F. *Invasive Physiotherapy.* 2nd ed. Madrid: Elsevier (2016).

[17] Gómez-Chiguano G Navarro-Santana M Cleland J Arias-Buría J Fernández-de-las-Peñas C Ortega-Santiago R Effectiveness of ultrasound-guided percutaneous electrolysis for musculoskeletal pain: a systematic review and meta-analysis. *Pain Med.* (2021) 22:1055–71. 10.1093/pm/pnaa342 33155055

[18] López-Royo M Ortiz-Lucas M Gómez-Trullén E Herrero P. The effectiveness of minimally invasive techniques in the treatment of patellar tendinopathy: a systematic review and meta-analysis of randomized controlled trials. *Evid Based Complement Alternat Med.* (2020) 2020:1–16. 10.1155/2020/8706283 32963575 PMC7492866

[19] Varela-Rodríguez S Cáceres-Pajuelo E Sánchez-Sánchez J. Percutaneous electrolysis in patients with musculoskeletal disorders: a systematic review. *J Mol Genet Med.* (2021) 15:476. 10.1016/j.jbmt.2018.05.002 30691739

[20] Augustyn D Paez A. The effectiveness of intratissue percutaneous electrolysis for the treatment of tendinopathy: a systematic review. *South Afr J Sports Med.* (2022) 34. 10.17159/2078-516X/2022/v34i1a12754 PMC9924571 36815929

[21] Moher D Shamseer L Clarke M Ghersi D Liberati A Petticrew M Preferred reporting items for systematic review and meta-analysis protocols (PRISMA-P) 2015 statement. *Rev Espanola Nutr Hum Diet.* (2016) 20:148–60. 10.1186/2046-4053-4-1 25554246 PMC4320440

[22] Higgins J Green S. *Cochrane Handbook for Systematic Reviews of Interventions Version 5.1. 0.* (2011).

[23] de Morton N. The PEDro scale is a valid measure of the methodological quality of clinical trials: a demographic study. *Aust J Physiother.* (2009) 55:129–33. 10.1016/S0004-9514(09)70043-1 19463084

[24] Cochran W. Some methods for strengthening the common χ 2 tests author(s). *Biometrics.* (1954) 10:417–51.

[25] Huedo-Medina T Sánchez-Meca J Marín-Martínez F Botella J. Assessing heterogeneity in meta-analysis: Q statistic or I2 index? *Psychol Methods.* (2006) 11:193–206. 10.1037/1082-989X.11.2.193 16784338

[26] Schunemann H Oxman A Brozek J Glasziou P Bossuyt P Chang S GRADE: assessing the quality of evidence for diagnostic recommendations. *Evid Based Med.* (2008) 13:162–3. 10.1136/ebm.13.6.162-a 19043023

[27] Austin T Richter R Sebelski C. Introduction to the GRADE approach for guideline development: considerations for physical therapist practice. *Phys Ther.* (2014) 94:1652–9. 10.2522/ptj.20130627 25035268

[28] Al-Boloushi Z Gómez-Trullén E Arian M Fernández D Herrero P Bellosta-López P. Comparing two dry needling interventions for plantar heel pain: a randomised controlled trial. *BMJ Open.* (2020) 10:e038033. 10.1136/bmjopen-2020-038033 32819949 PMC7440826

[29] de la Barra Ortiz H Cancino J Peña F León F Donoso E Gaete V. Effectiveness of percutaneous microelectrolysis and ultrasound in decreasing pain in myofascial trigger points: evaluation through algometry and visual analogue scale. *Physiother Q.* (2020) 28:1–8. 10.5114/PQ.2020.95768

[30] Arias-Buría J Truyols-Domínguez S Valero-Alcaide R Salom-Moreno J Atín-Arratibel M Fernández-de-las-Peñas C. Ultrasound-guided percutaneous electrolysis and eccentric exercises for subacromial pain syndrome: a randomized clinical trial. *Evid Based Complement Alternat Med.* (2015) 2015:1–9. 10.1155/2015/315219 26649058 PMC4662984

[31] Rodríguez-Huguet M Góngora-Rodríguez J Rodríguez-Huguet P Ibañez-Vera A Rodríguez-Almagro D Martín-Valero R Effectiveness of percutaneous electrolysis in supraspinatus tendinopathy: a single-blinded randomized controlled trial. *J Clin Med.* (2020) 9:1837. 10.3390/jcm9061837 32545583 PMC7356532

[32] de Miguel Valtierra L Salom Moreno J Fernández-de-las-Peñas C Cleland J Arias-Buría J. Ultrasound-guided application of percutaneous electrolysis as an adjunct to exercise and manual therapy for subacromial pain syndrome: a randomized clinical trial. *J Pain.* (2018) 19:1201–10. 10.1016/j.jpain.2018.04.017 29777953

[33] Moreno M. Results of the electrolysis percutaneous intratissue in the shoulder pain: infraspinatus, a randomized controlled trial. *Rev Cubana Ortop Traumatol.* (2015) 29:76–87.

[34] Moreno C Mattiussi G Núñez F Messina G Rejc E. Intratissue percutaneous electolysis combined with active physical therapy for the treatment of adductor longus enthesopathy-related groin pain: a randomized trial. *J Sports Med Phys Fitness.* (2017) 57:1318–29. 10.23736/S0022-4707.16.06466-5 28116876

[35] Rodríguez-Huguet M Góngora-Rodríguez J Lomas-Vega R Martín-Valero R Díaz-Fernández Á Obrero-Gaitán E percutaneous electrolysis in the treatment of lateral epicondylalgia: a single-blind randomized controlled trial. *J Clin Med.* (2020) 9:2068. 10.3390/jcm9072068 32630241 PMC7408752

[36] López-Royo M Ríos-Díaz J Galán-Díaz R Herrero P Gómez-Trullén EM. A. Comparative study of treatment interventions for patellar tendinopathy: a randomized controlled trial. *Arch Phys Med Rehabil.* (2021) 102:967–75. 10.1016/j.apmr.2021.01.073 33556350

[37] Lopez-Martos R Gonzalez-Perez L Ruiz-Canela-Mendez P Urresti-Lopez F Gutierrez-Perez J Infante-Cossio P. Randomized, double-blind study comparing percutaneous electrolysis and dry needling for the management of temporomandibular myofascial pain. *Med Oral Patol Oral Cir Bucal.* (2018) 23:e454–62. 10.4317/medoral.22488 29924769 PMC6051683

[38] De-la-Cruz-Torres B Barrera-García-Martín I Valera-Garrido F Minaya-Muñoz F Romero-Morales C. Ultrasound-guided percutaneous needle electrolysis in dancers with chronic soleus injury: a randomized clinical trial. *Evid Based Complement Alternat Med.* (2020) 2020:1–8. 10.1155/2020/4156258 32908559 PMC7474345

[39] Martínez-Silván D Santomé-Martínez F Champón-Chekroun A Velázquez-Saornil J Gómez-Merino S Cos-Morera M Clinical use of percutaneous needle electrolysis in musculoskeletal injuries: a critical and systematic review of the literature. *Apunts Sports Med.* (2022) 57:100396. 10.1016/j.apunsm.2022.100396

[40] Asensio-Olea L Leirós-Rodríguez R Marqués-Sánchez M de Carvalho F Maciel L. Efficacy of percutaneous electrolysis for the treatment of tendinopathies: a systematic review and meta-analysis. *Clin Rehabil.* (2022):026921552211442. 10.1177/02692155221144272 36583575

[41] Salaffi F Stancati A Silvestri C Ciapetti A Grassi W. Minimal clinically important changes in chronic musculoskeletal pain intensity measured on a numerical rating scale. *Eur J Pain.* (2004) 8:283–91. 10.1016/j.ejpain.2003.09.004 15207508

[42] Valera-Calero J Sánchez-Mayoral-Martín A Varol U. Short-term effectiveness of high- and low-intensity percutaneous electrolysis in patients with patellofemoral pain syndrome: a pilot study. *World J Orthop.* (2021) 12:781–90. 10.5312/wjo.v12.i10.781 34754834 PMC8554351

[43] Varela-Rodríguez S Sánchez-Sánchez J Velasco E Delicado-Miralles M Sánchez-González J. Endogenous pain modulation in response to a single session of percutaneous electrolysis in healthy population: a double-blinded randomized clinical trial. *J Clin Med.* (2022) 11:2889. 10.3390/jcm11102889 35629015 PMC9143044

[44] Lv Z Shen L Zhu B Zhang Z Ma C Huang G Effects of intensity of electroacupuncture on chronic pain in patients with knee osteoarthritis: a randomized controlled trial. *Arthritis Res Ther.* (2019) 21:120. 10.1186/s13075-019-1899-6 31088511 PMC6518678

