# Supplementary figures and images for: Efficacy of different intensities of percutaneous electrolysis for musculoskeletal pain: A systematic review and meta-analysis
Source: Front Med (Lausanne). 2023 Feb 2;10:1101447. doi: 10.3389/fmed.2023.1101447 (PMC9932994; doi:10.3389/fmed.2023.1101447)

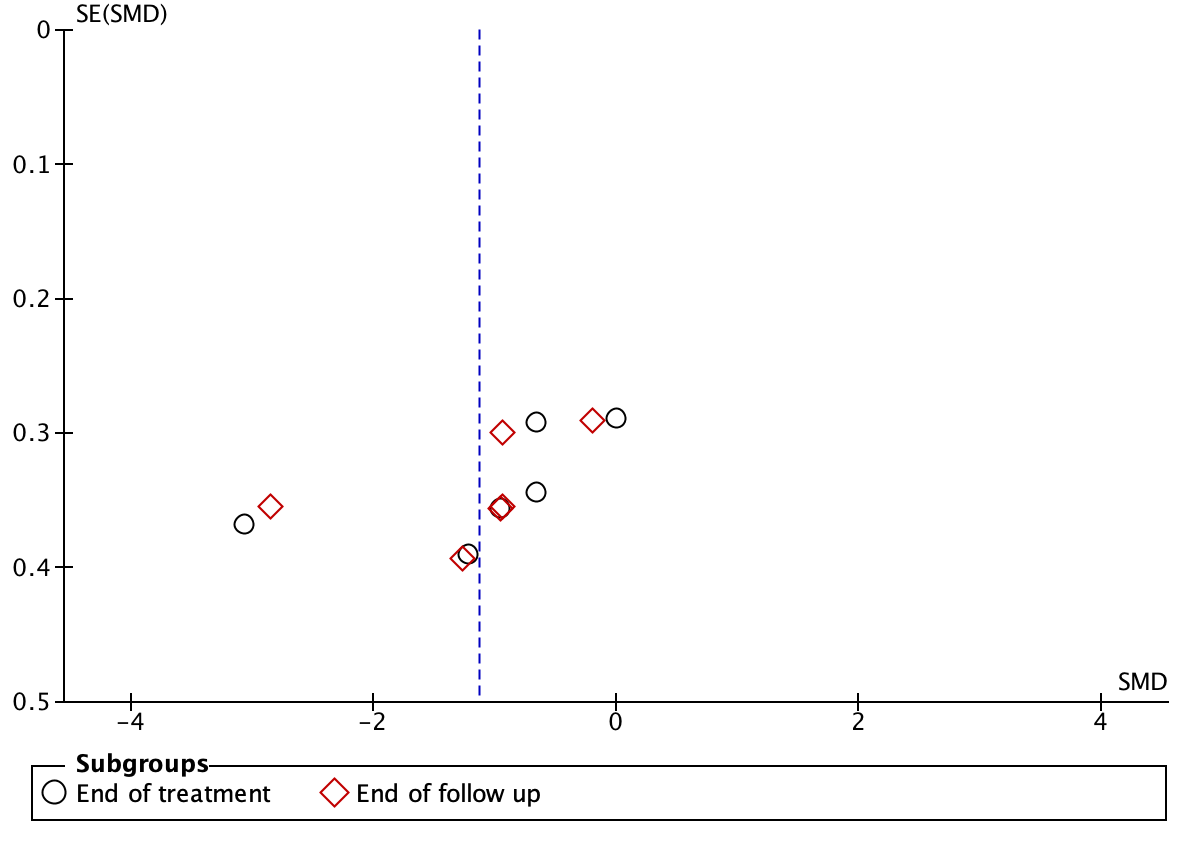

Supplement: Supplementary Figure 1 — Funnel low. [file Image_1.PNG]

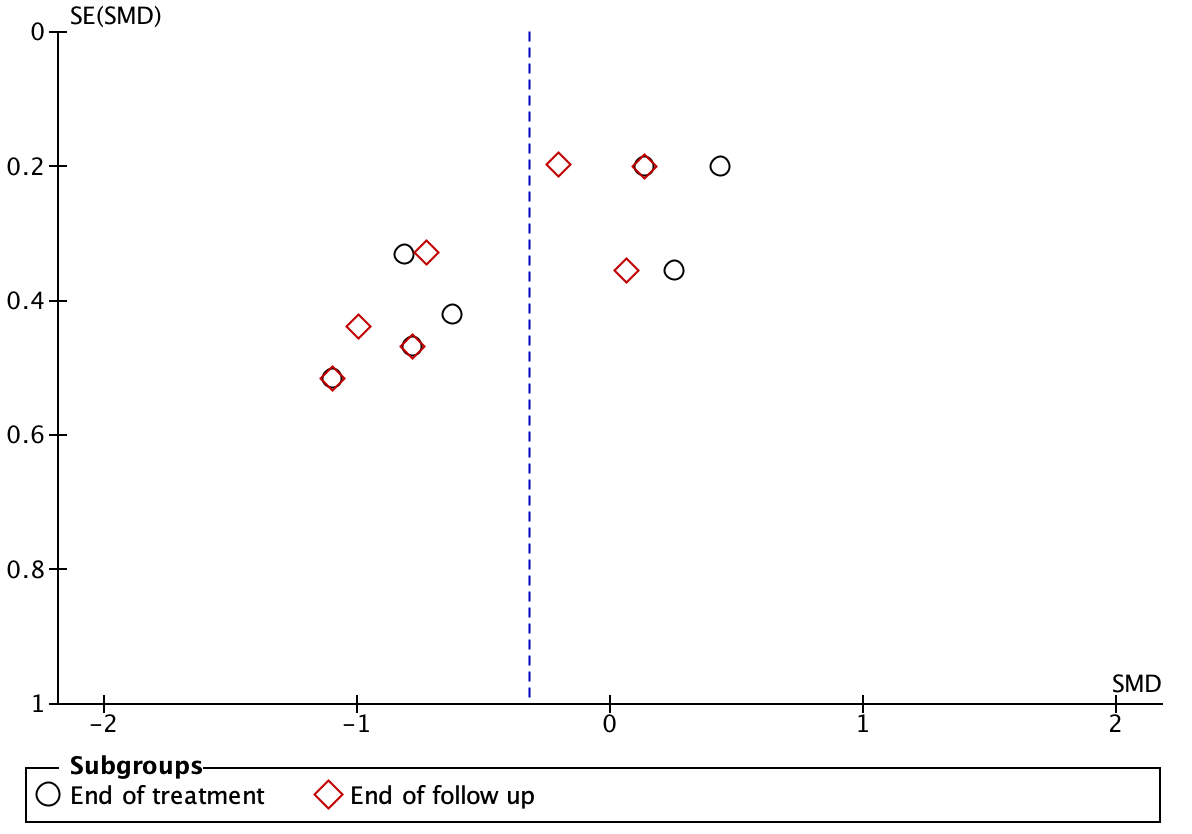

Supplement: Supplementary Figure 2 — Funnel high. [file Image_2.PNG]
