# Supplementary material for: Efficacy of different intensities of percutaneous electrolysis for musculoskeletal pain: A systematic review and meta-analysis
Source: Front Med (Lausanne). 2023 Feb 2;10:1101447. doi: 10.3389/fmed.2023.1101447 (PMC9932994; doi:10.3389/fmed.2023.1101447)
Supplement: Supplementary Table 1 — Parameters of percutaneous electrolysis interventions of the included trials. [file Table_1.pdf]

Supplementary Table 1: Parameters of Percutaneous Electrolysis Interventions of the Included Trials

Study	Group	No. punctures	Needle approach	Gauge (mm)	Depth (mm)	Time of electrical current	Intensity of the electrical current	Device
**High Intensity (>1 mA)**								
Al-Boulishi et al, 2019 (27)	G1	4	Soleus, gastrocnemius, quadratus plantae, flexor digitorum brevis and abductor hallucis TrPs according to the technique described by Travell and Simmons	0.25-0.30	Depending on anthropometry of the subject	NR	1.5 mA	Physio Invasiva®, PRIM Fisioterapia, Spain.
Moreno et al, 2015 (32)	G2	NR	All detected TrPs	Depending on anthropometry of the subject	Depending on anthropometry of the subject	4 seconds x 3 schocks	6 mA	EPI Advanced Medicine®, Barcelona, Spain
	G3	NR	Infraspinatus tendon					
	G4	NR	All detected TrPs and infraspinatus tendon					
Moreno et al. 2017 (33)	G1	6	The needle acting as cathode was applied over the enthesis of the adductor longus. The technique was US-guided	0.33	50	Three applications (three right and three left) of 5seconds each	3mA	EPI Advanced Medicine®, Barcelona, Spain
García-Naranjo et al. 2017 (12)	G1	1	The needle acting as cathode targeted the scapular insertion of the levator scapule.	0.16	25	Three schocks of 3 seconds with a resting interval of 1-2 minutes	2-4mA, the intensity was increased 1mA/sec	Physio Invasiva®

			The technique was US-guided					
López – Martos et al, 2018 (36)	G1	3	The needle acting as cathode was applied in the Lateral Pterigoid Muscle, according to the technique described by Koole et al.	0.25	40	three times for 3 seconds,	2 mA	EPI Advanced Medicine®, Barcelona, Spain
Cruz-Torres et al, 2020 (37)	G1<br>G3	2<br>2 + exercise	The needle acting as cathode was applied in central tendon of the soleus. The technique was US-guided	0.30	40	three times for 3 seconds	2.5mA	Physio Invasiva®, PRIM Fisioterapia, Spain.
López-Royo et al, 2021 (35)	G1	4	The needle acting as cathode was applied in the Patellar tendon. The technique was US-guided	0.25	25	three times for 3 seocnds	3mA	NR

**Supplementary Table 2:** Parameters of Percutaneous Electrolysis Interventions of the Included Trials								
**Study**	**Group**	**No. punctures**	**Needle approach**	**Gauge (mm)**	**Depth (mm)**	**Time of electrical current**	**Intensity of the electrical current**	**Device**
**Low Intensity (<1 mA)**								
Fernández Rodríguez et al, 2018 (13)	G1	1	The needle acting as cathode targeted the the proximal plantar fascia at the medial calcaneal tubercle. The technique was US-guided	0.35	40	NR	28 mC	EPTE v2; Ionclinics, Valencia, Spain
Rodríguez-Huguet et al. 2020A (30)	G1	1	The needle acting as cathode targeted the insertional tendon of the muscles of the epicondyle. The technique was US-guided.	0.3	NR	1.2 min	350 μA	EPTE®, Ionclinics, Valencia, Spain
Rodríguez-Huguet et al. 2020B (34)	G1	1	The needle acting as cathode targeted the supraspinatus tendon. The technique was US-guided.	NR	NR	1.2 min	350 μA	EPTE®, Ionclinics, Valencia, Spain
Miguel-Valtierra et al. 2018 (31)	G1	1	The needle acting as cathode targeted the supraspinatus tendon. The technique was US-guided.	0.3	25	90 seconds	350 μA	EPTE® V01, classification IIa, Ionclinics, Valencia, Spain

Arias-Buría et al. 2015 (29)	G1	1	The needle acting as cathode targeted the supraspinatus tendon. The technique was US-guided.	0.3	25	1.2 minutes	350 μA	EPTE® V01, classification IIa, Ionclinics, Valencia, Spain
Barra-Ortiz et al, 2020 (28)	G1	1	The needle acting as cathode targeted the Short trapezius muscle.	NR	NR	until the patience feel pain, 3 times with 30 seconds of pause	600 μA	Sveltia DC equipment
